# The Control of Hydrophobia

**Published:** 1919-07-26

**Authors:** 


### THE CONTROL OF HYDROPHOBIA.

### A More Simplified Procedure Necessary.

Coincident with a number of complaining letters in the daily Press from persons bitten by suspected animals come the re-issue and revision of the Memorandum on the procedure to be adopted in these cases. The arrangements made and now to be carried out by the Ministry of Health concern chiefly the local medical officer of health, but the process whereby the details of the case come to his knowledge appear to be of a most unnecessarily complicated nature. The sufferer is advised to communicate at once with the police, who will make the "necessary inquiries" and will also notify the Board of Agriculture and Fisheries, which Board then passes on the information to the medical officer of health, who must at once report all possible details concerning the dog and the incidence of the disease in his district to the Medical Department of the Ministry of Health.

Thus ends the first stage of this long line of communication; but still it is far from complete. The Ministry nexts proceeds to ascertain whether the officers of the Board of Agriculture and Fisheries consider, from the facts now in official possession, that the symptoms in the dog are sufficient to justify anti-rabic treatment without awaiting a pathological diagnosis; whether Negri bodies have been found in the animal's brain, and whether the animal is to be considered rabid. This information, when received, is handed back to the local medical officer, who must, however, in the event of a positive diagnosis in the dog and a possible human infection, await instructions from the Health Ministry before sending cases up for treatment. Such is an outline of this new routine, and although we understand that the Board of Agriculture is authorised to communicate directly with the medical officer of health if it learns of a suspected case of rabies—certainly a possible step towards simplification—yet, in view of the fact that final decision whether or no anti-rabic treatment is necessary in any individual rests with the medical officer himself, it seems that the obvious and most efficient procedure would have been for the patient and any general practitioner concerned with the case to communicate at once and directly with the local health department. It is well known that the time factor is of paramount importance with regard to anti-rabies treatment, and surely such a complicated interchange of details and such divided control must inevitably hinder, if not completely defeat, satisfactory working of preventive schemes.

Now that the matter is placed in the hands of the medical officer of health, who has, usually, considerable facilities to deal with the practical side of the diagnosis, we suggest it would be far better to have taken this opportunity to cut down, rather than increase, the complexities of his relations with the authorities. Treatment centres are now working in London, Plymouth, Cardiff, Birmingham, Manchester, and Newcastle, but the hospitals concerned do not guarantee to provide accommodation for patients undergoing the necessary two or three weeks' course. At once the difficulty of providing funds for living during this period arises. Here again delay must occur, for in the case of poorer patients the medical officer can only represent the matter to the District Council, in order that the requisite money be found. Well may the Memorandum urge that all possible expedition be secured in the above procedure.

